# Internet Addiction and Sleep Disorders among Medical Students

**DOI:** 10.1155/2023/6685676

**Published:** 2023-09-15

**Authors:** Seyyed Mansour Kashfi, Hamed Karami, Fatemeh Jafari, Masumeh Daliri, Maryam Yazdankhah, Amirhossein Kamyab, Ali Khani Jeihooni

**Affiliations:** ^1^Department of Public Health, School of Health, Shiraz University of Medical Sciences, Shiraz, Fars, Iran; ^2^Student Research Committee, Shiraz University of Medical Sciences, Shiraz, Iran; ^3^Department of Public Health, Qaen Faculty of Medical Sciences, Birjand University of Medical Sciences, Birjand, Iran; ^4^Department of Community Medicine, School of Medicine, Fasa University of Medical Sciences, Fasa, Iran; ^5^Nutrition Research Center, Department of Public Health, School of Health, Shiraz University of Medical Sciences, Shiraz, Iran

## Abstract

**Background:**

Considering the increasing use of the Internet in Iranian society, especially among students, and the importance of sleep quality, the present study investigated the relationship between sleep quality and Internet addiction among medical students in Shiraz.

**Methods:**

In this descriptive-analytical study, the sample included students of the Shiraz University of Medical Sciences who were selected by a multistage sampling method in 2018. Each faculty was considered to be stratified, and the samples were selected from all strata by simple random sampling. A total of 400 student questionnaires were eligible for analysis. The level of sleep disturbance was measured using the Pittsburgh Sleep Quality Index (PSQI), and Young's Internet Addiction Test (IAT) was used to evaluate Internet addiction. Cronbach's alpha for PSQI and IAT was 0.77 and 0.93, respectively.

**Results:**

109 (%28.9) and 58 (%14.4) of all the people under study were at risk of Internet addiction or poor sleep quality, respectively. The highest percentage of poor sleep quality was in those who were addicted to the Internet (60%), but the lowest percentage was observed in the group without addiction (27%). In addition, there was a significant correlation between Internet addiction and subjective sleep quality (*r* = 0.191, *p* = 0.05), sleep latency (*r* = 0.129, *p* = 0.01), sleep duration (*r* = 0.119, *p* = 0.01), habitual sleep efficiency (*r* = 0.186, *p* = 0.05), sleep disturbances (*r* = 0.169, *p* = 0.01), use of sleeping medication (*r* = 0.203, *p* = 0.05), and daytime dysfunction (*r* = 0.188, *p* = 0.05).

**Conclusion:**

These findings help national health officials and planners in Iran to design appropriate and effective interventions to improve students' health and prevent Internet addiction.

## 1. Background

In recent years, the Internet has explosively grown and transformed the world. Better opportunities for education, communication, banking, business, health seeking, and social interaction have been provided through the Internet [1]. Iran has also experienced a huge rise in Internet usage, with young people making up the majority of users [2]. Internet usage has its pros and cons as well. On the one hand, it can be useful and effective; on the contrary, it can be harmful and cause mental health problems [3].

Yang coined the phrase “Internet addiction,” which has since attracted the attention of psychologists, psychiatrists, therapists, scientists, and especially Internet users [4]. Internet addiction is defined as excessive or uncontrolled preoccupations, urges, or behaviors regarding utilizing and accessing the Internet that might result in a disorder [5]. Increased use of digital media and the Internet mildly affects people's sleep habits, and with changes in sleep quality, symptoms of depression, anxiety, and stress are exacerbated [6]. Internet addiction is effective on the quality and quantity of sleep of medical students, especially in men, due to more use of social networks, and it leads to their daily dysfunction [7]. Also, this addiction is associated with signs of social exclusion and other prevalent psychiatric problems such as mood disorders, anxiety, poor impulse control, and substance abuse [5]. The prevalence of Internet addiction varies around the world, and teenagers and young people are more likely to succumb to it [8].

In a meta-analysis conducted by Salarvand et al., the overall prevalence of Internet addiction was 31.51% among Iranian students [9]. Excessive Internet use has been linked to a variety of sleep-related problems, including poor sleep quality, extended sleep latency, short sleep durations, and sleep disruptions. It has also been shown to increase the use of sleeping pills and have negative effects on everyday functioning [10]. In another meta-analysis including 35,684 participants, the overall odds ratio of sleep disorders caused by Internet addiction was calculated to be 2.20 [11]. Internet users lacking self-control are more likely to suffer from poor sleep quality as a result of their uncontrolled online behavior (such as staying up late) [10]. In a former study conducted on a population of medical students, the prevalence of poor sleep quality was 61.7%, which was associated with daily life behaviors, especially excessive Internet usage [12]. Epidemiological studies have shown that sleep disturbance is associated with adverse health outcomes, including obesity, type 2 diabetes, cardiovascular disease, hypertension, and premature death [13]. In general, the relationship between Internet addiction and sleep quality has been shown in various studies. But other psychosocial characteristics which aggravate Internet addiction is less considered [7, 14]. Systematic reviews and meta-analyses have provided a significant understanding *t* into the relationship between Internet addiction and sleep quality and disorders [9, 11]. One of the limitations of cross-sectional studies is the choice of study courses to conduct the study, which does not reflect the state of Internet use and the sleep quality of the participants throughout the year [7, 10].

The effect of Internet addiction on sleep quality and the correlation between Internet use and sleep disorders among college students needs more attention. Considering the increase in the use of the Internet in Iran, especially among students, the relationship between Internet addiction and sleep disorders, and the importance of this issue to minimize the inappropriate use of the Internet, the present study aims to determine the effect of Internet addiction and sleep and communication disorders. These two were designed among the students of the Shiraz University of Medical Sciences. This study generally examines these two issues: comparison of sleep quality between participants with Internet addiction and correlations between dimensions of sleep quality and Internet addiction.

## 2. Materials and Methods

### 2.1. Study Design and Population

This study was conducted on students studying at the Shiraz University of Medical Sciences in 2018. Due to the fact that exposure (Internet addiction) and outcome (sleep disorder) information were taken from the subjects at the same time, the type of study was cross-sectional. In [15], the sample size based on formula *n* = *z*^2^pq/*d*^2^ (*p* = 0.26, *d* = 0.05) was estimated to be 323 people and more than 400 (402 participants), according to attrition. The sample size was selected from nine relevant faculties using a stratified random sampling method.

In order to generalize the results, samples should have been selected from all university students. Therefore, a multistage sampling method was used. First, the proportion of the students of each faculty to the total number of university students was determined. Then, by multiplying the obtained proportion by the sample size of the research, the proportion of each faculty was determined. Each faculty was considered to be stratified, and we conducted simple random sampling in all strata. Then, in the third stage, the selected people were confirmed if they met the eligibility criteria. Finally, the questionnaires were completed by referring to the students' classrooms.

### 2.2. Inclusion and Exclusion Criteria

Inclusion criteria were as follows:

- Willingness to participate in the study
- Completing the questionnaires correctly and completely
- Regular use of the Internet at least during the past year
- Completing at least one semester of the entire study period at the university

Exclusion criteria were as follows:

- Diagnosis of psychiatric disorders
- Absence during the study period
- Incomplete questionnaire completion
- Lack of interest or informed consent to participate in the study

### 2.3. Measures

The data collection tools used in this study were a demographic information questionnaire, Pittsburgh Sleep Quality Questionnaire (PSQI), and Kimberly Young Internet Addiction Questionnaire (IAT). The demographic information questionnaire included age, gender, place of residence, marital status, level of education, average grade, father's education, mother's education, and job status of the participants.

#### 2.3.1. Pittsburgh Sleep Quality Questionnaire (PSQI)

PSQI is a self-report questionnaire developed by Buysse, which appraises sleep quality through a standardized questionnaire, and can be easily understood and answered, differentiating between “good sleepers” and “poor sleepers.” It assesses sleep quality and quantity over a 1-month time interval. This questionnaire has seven scales examining the subjective sleep quality, sleep latency, sleep duration, habitual sleep efficiency, sleep disturbances, use of sleeping medications, and daytime dysfunction. The questions related to each dimension were scored from zero to three: score zero indicated no sleep disturbance, score one showed moderate sleep disturbance, score two was related to severe sleep disturbance, and score three showed very serious sleep disturbance. The total score was measured by summing the scores of seven dimensions (range, 0–21), and a total score higher than five indicated poor sleep quality [16]. The Iranian version of this questionnaire has been confirmed by Moghaddam et al. Cronbach's alpha coefficient was calculated to be 0.77, indicating the internal consistency of the questionnaire [17].

#### 2.3.2. Kimberly Young Internet Addiction Questionnaire (IAT)

This questionnaire, which was prepared by Kimberly Young, contains 20 questions on a five-point Likert scale to measure people's dependence on the Internet, ranging from 1 (rarely) to 5 (always), and was scored between 20 and 100. Based on the scores obtained from this scale, people are divided into three groups, including no Internet addiction (scores from 20 to 49), at risk of Internet addiction (scores from 50 to 79), and having Internet addiction (scores from 80 to 100) [4]. The reliability and validity of this questionnaire have already been confirmed by Cronbach's alpha coefficient of 0.93 [18]. Also, in the study conducted in Iran, the content validity index for each item was higher than 0.83 and the average content validity index was equal to 0.89. Face validity was measured using the item importance index, and this index demonstrated that all items have an importance index higher than 1.5 [19].

### 2.4. Statistical Analysis

The collected data were analysed using SPSS version 26 software. Qualitative data were presented as frequency and percentage, and quantitative data were presented as mean and standard deviation. Univariate analysis was performed using the chi-square test for the relationship between two or more categorical variables (demographic characteristics and sleep quality and Internet addiction), the independent *t*-test for the relationship between a quantitative variable and two-state qualitative variable (age and average grade and sleep quality), and the analysis of variance for the relationship between a quantitative variable and three-state qualitative variable (comparison of the score of sleep quality dimensions in different groups of Internet addiction). The correlation between Internet addiction and all dimensions of sleep quality was calculated using the Pearson correlation test, and *p* value <0.05 was considered statistically significant.

### 2.5. Ethical Approval and Consent to Participate

Ethical approval was obtained from the Human Research Ethics Committee at the Shiraz University of Medical Sciences (Ethical Code: IR.SUMS.REC.197.914, https://ethics.research.ac.ir/EthicsProposalView.php?&code=IR.SUMS.REC.1397.914). All study participants provided written informed consent. Permission was also obtained to digitally record all interviews.

## 3. Results

Out of 402 participants, 245 (60.9%) were female and 353 (87.8%) were single. Ph.D. and professional doctorate were the most common grades (49.3%), and 255 (63.4%) did not go to work (Table 1).

109 participants (28.9%) were at risk for or had Internet addiction. The average age of Internet addicts (27.66 ± 11.59) was higher than that of other groups, while the average grade of this group was lower than that of other groups (15.5 ± 2.32), which was not statistically significant (*p* = 0.206). Male gender, single participants, and Ph.D. and professional doctorate degrees were the most people at risk for or addicted to the Internet (Table 1).

Overall, 58 (14.4%) participants had poor sleep quality, of which 35 (60.3%) were women, 52 (89.7%) lived in urban areas, and most of them had university-educated parents. Moreover, the average age and grade point average of those with poor sleep quality were lower (21.96 ± 2.71 and 16.2 ± 1.5, respectively) than those with good sleep quality, but it was not statistically significant. Table 1 provides further demographic information.

According to Table 2, the average score of sleep quality in those with Internet addiction was higher than that of other compared groups (*p* = 0.026). The average score of subjective sleep quality (*p* = 0.04), sleep disturbances (*p* = 0.005), and the use of sleeping medication (*p* < 0.001) was significantly higher in the group at risk of Internet addiction. Moreover, in those who had Internet addiction, the average score of habitual sleep efficiency (*p* = 0.037) was significantly higher than that of those at risk or without Internet addiction.

**Figure 1:**
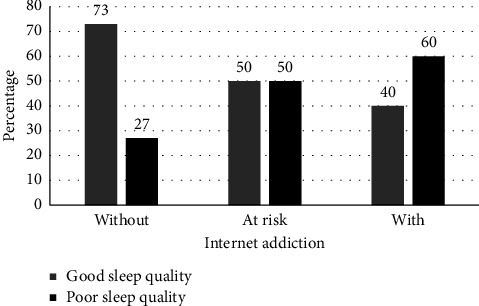
Percentage of students at levels of sleep quality among the three levels of Internet addiction.

The results of this study revealed that the highest percentage of poor sleep quality was in those who are addicted to the Internet (60%), while the lowest percentage was observed in the group without addiction (27%), and in the at-risk group, poor sleep quality and good sleep quality were equal (50%) (Figure 1).

As given in Table 3, there is a significant correlation between Internet addiction and subjective sleep quality (*r* = 0.191, *p* = 0.05), sleep latency (*r* = 0.129, *p* = 0.01), sleep duration (*r* = 0.119, *p* = 0.01), habitual sleep efficiency (*r* = 0.186, *p* = 0.05), sleep disturbances (*r* = 0.169, *p* = 0.01), use of sleeping medication (*r* = 0.203, *p* = 0.05), and daytime dysfunction (*r* = 0.188, *p* = 0.05).

## 4. Discussion

Adequate sleep is essential for proper body function. With the increasing popularity of smart phones and the increasing use of the Internet, especially among the youth, using them before bedtime has become a habit, which can negatively affect their quality of sleep [10]. Therefore, the present study was conducted with the aim of determining the relationship between Internet addiction and sleep quality in a population of university students.

Based on the results of our study, 28.9% of all the people under study were at risk of Internet addiction or had Internet addiction, and this result is higher than the rates obtained from Vietnam University (21.2%) [20] and lower than results of other countries such as Bangladesh (49.2%) and Jordan (40.0%) [21, 22]. The prevalence of poor sleep quality found in this study is 14.4% which is lower than that of other studies in Iran and Bangladesh [15, 21], and the highest percentage of poor sleep quality was witnessed in those addicted to the Internet (60%). Other studies have reported this amount from 26.7% to 95% in severe Internet addiction [20, 21]. Despite the lower prevalence of Internet addiction and sleep disorder in this study compared to other studies, however, the high prevalence of poor sleep quality in people addicted to the Internet in this study is consistent with the aforementioned studies, and these results can confirm that Internet addiction is known as one of the factors affecting the low quality of sleep; on the other hand, changes in the prevalence of Internet addiction among different countries may be due to differences in evaluation methods as well as cultural and social factors.

### 4.1. Association between Demographic Characteristics, Internet Addiction, and Sleep Quality

Derived from our results, there was no significant difference between the age and gender variables with sleep quality and Internet addiction, which was consistent with the results of the studies by Gupta et al. [23] and Sağar and Hülya [24]. However, other studies have demonstrated that Internet addiction was more prevalent among men at a young age and did not have significant gender differences in sleep quality [25, 26]. Although there are age-related differences in sleep duration and quality, as well as Internet usage, the fact that the university students in this study were in the same age group may have influenced this result. In addition, considering the necessity of sleep for the survival of any living thing in this context, the fact that sleep is not a gender-specific variable may reveal the result of this study [24].

The present study found no significant effect of the place of residence, level of education, and marital status on Internet addiction and sleep quality of the students. Consistently, the study by Tahir et al. [1] found no significant effect of residence on Internet addiction but stated that participants who lived in urban areas had poorer sleep quality than those who lived in rural areas. Our study was not in line with the results of studies by Romero Blanco et al. [25] and Cheng et al. [27] who found that undergraduate students had poorer sleep quality than graduate students. The study by Zhang et al. [20] stated that single people were not at risk of sleep-related problems. The study by Karimi et al. [28] also noted that the rate of Internet addiction was higher among single students, and these results were not consistent with those of our study. Most of the people studied were single, which probably affected the results of the study.

### 4.2. Internet Addiction and Sleep Quality

A comparison of the scores of sleep quality between participants without Internet addiction and those with different degrees of Internet addiction revealed the mean score of the Global PSQI index was higher in at-risk participants of Internet addiction and Internet addicts than in other participants; this result was also maintained in the dimensions of subjective sleep quality, habitual sleep efficiency, sleep disturbances, and use of sleeping medication. This finding was consistent with the results of a study in southern Taiwan and a multinational cross-sectional survey that was carried out in seven countries. These studies introduced various components of Internet addiction as significant predictors of subjective sleep quality, sleep disturbance, use of sleep medication, sleep duration, daytime dysfunction, and sleep latency [1, 10]. In general, some studies have also stated Internet addiction as a predictor of sleep quality of students, children, and teenagers [29, 30]. So, a study stated that Internet addiction significantly affects the sleep quality of students, and with an increase of 1 unit in the Internet addiction score, the risk of low sleep quality increases by 8.7% [24]. This finding could be due to the mechanisms related to the use of electronic devices in bed harming sleep with cognitive, emotional, or physiological stimulation; it can also be said that exposure to intense light leads to the suppression of melatonin secretion and sleep delay, which can increase consciousness and sleep disorders [11, 31].

### 4.3. Correlation between Internet Addiction and Sleep Quality

Also, the findings of our study showed that there was a positive week correlation between Internet addiction and subjective sleep quality, sleep latency, sleep disturbances, sleep duration, daytime dysfunction, habitual sleep efficiency, use of sleeping medication, and the global PSQI; this finding was consistent with that of studies conducted in Taiwan [10], Saudi Arabia [32], and South Asia [33].

Regarding long sleep latency and Internet addiction, it can be noted that stimulation of the central nervous system through games, watching online movies before sleep, and the emission of blue light through the screen can lead to the suppression of melatonin secretion from the well-known pineal gland, which, in turn, can be an effective factor in long-term sleep latency [34, 35].

Studies conducted in South Korea [36] and China [37] on the link between Internet use and sleep duration have identified a significant negative association between Internet use and sleep duration, which are not consistent with our results. This can be because staying awake at night and spending time on the Internet lead to a longer sleep duration during the day. In addition, this result can lead to disruption of normal daily functions.

The identification of a positive correlation between Internet addiction and the use of sleep medication in this study and other studies with similar results [1, 10] may imply that one of the most common ways to tackle sleep disorders is the use of sleep medications; with Internet addiction and increased sleep disorder, the tendency to take medical sleep medications increases [10, 38].

### 4.4. Strengths and Limitations

The strengths of the study can be mentioned as follows:

- Its appropriate sample size
- Examining the prevalence of Internet addiction
- Using a valid questionnaire as an index of Pittsburgh sleep quality to determine the severity of sleep disorder
- In addition, students of different educational levels were included in the study, so a significant proportion of participants were masters' and Ph.D. students and professional doctorates.

The limitations of the study can be mentioned as follows.

Despite the strength of our research, this study was a cross-sectional study, and therefore, the results do not indicate a causal relationship; we have only been able to clarify the relationship between Internet addiction and sleep quality, but due to our research methodology, we did not determine its cause and effect. In addition, since the sample includes students from one university, the results cannot be considered representative of the entire country.

## 5. Conclusion

The results of the research show that Internet addiction is considerably correlated with different aspects of sleep quality in students. Considering the significant role of students in the future as professionals, it is highly advised that educational programs and workshops about harms of Internet addiction be provided to students via social media and other platforms. Therefore, according to the results of this study, which is a confirmation for the results of similar studies, it is recommended that health service providers should provide the necessary awareness in this field to the public, including students' parents. Also, health policymakers and the media should provide the necessary plans to inform people through booklets and brochures.

### 5.1. Recommendations for Future Studies

Based on this, it is suggested that future studies that investigate the relationship between Internet addiction and sleep quality should be conducted with the participation of people in different age groups. Also, future studies can be expanded by adding different variables to this field and investigating different aspects. In addition, it is suggested that studies be designed to examine the causality between sleep quality and Internet addiction.

## Figures and Tables

**Table 1:** Demographic characteristics of the students based on their sleep quality and Internet addiction.

Variables			Internet addiction				Sleep quality		
			No Internet addiction	At risk of Internet addiction	With Internet addiction	*p* value	Good	Poor	*p* value
			276 (68.7%)	108 (26.9%)	8 (2%)		111 (27.6%)	58 (14.4%)	
Age (mean ± SD)	21.79 ± 3.35		21.87 ± 3.36	21.56 ± 2.73	27.66 ± 11.59	0.591	23.3 ± 4.45	21.96 ± 2.71	0.143

Gender	Male	157 (40%)	108 (39.3%)	40 (37%)	4 (50%)	0.745	40 (36%)	23 (39.7%)	0.644
	Female	245 (60.9%)	167 (60.7%)	68 (63%)	4 (50%)		71 (64%)	35 (60.3%)	

Living place	Urban	360 (89.6%)	247 (89.5%)	96 (88.9%)	7 (87.5%)	0.972	102 (91.9%)	52 (89.7%)	0.627
	Rural	42 (10.4%)	29 (10.5%)	12 (11.1%)	1 (12.5%)		9 (8.1%)	6 (10.3%)	

Marital status	Single	353 (87.8%)	237 (85.9%)	99 (91.7%)	7 (87.5%)	0.303	91 (82%)	54 (93.1%)	0.049
	Married	49 (12.2%)	39 (14.1%)	9 (8.3%)	1 (12.5%)		20 (18%)	4 (6.9%)	

Level of education	Bachelor	168 (41.8%)	112 (40.6%)	49 (45.4%)	4 (50%)	0.799	36 (32.4%)	16 (27.6%)	0.511
	Master of science	36 (9%)	27 (9.8%)	9 (8.3%)	0		14 (12.6%)	5 (8.6%)	
	Ph.D. and professional doctorate	198 (49.3%)	137 (49.6%)	50 (46.3%)	4 (50%)		61 (55%)	37 (63.8%)	

Average grade (mean ± SD)		16.38 ± 1.87	16.39 ± 1.85	16.58 ± 1.38	15.5 ± 2.32	0.206	16.57 ± 2.01	16.2 ± 1.5	0.219

Father's education	<12 years	47 (11.7%)	31 (11.2%)	14 (13%)	0	0.598	17 (15.3%)	8 (13.8%)	0.888
	High school diploma	107 (26.6%)	70 (25.4%)	33 (30.6%)	2 (25%)		27 (24.3%)	16 (27.6%)	
	Academic	248 (61.7%)	175 (63.4)	61 (56.5%)	6 (75%)		67 (60.4%)	34 (58.6%)	

Mother's education	<12 years	84 (20.9%)	60 (21.7%)	21 (19.4%)	0	0.473	27 (24.3%)	10 (17.2%)	0.263
	High school diploma	119 (29.6%)	84 (30.4%)	30 (27.8%)	4 (50%)		39 (35.1%)	17 (29.3%)	
	Academic	199 (49.5%)	132 (47.8%)	57 (52.8%)	4 (50%)		45 (40.5%)	31 (53.4%)	

Job	Unemployed	255 (63.4%)	181 (65.6%)	62 (57.4%)	3 (37.5%)	0.002	68 (61.3%)	37 (63.8%)	0.892
	Part-time	136 (33.8%)	89 (32.2%)	43 (39.8%)	3 (37.5%)		40 (36%)	19 (32.8%)	
	Full-time	11 (2.7%)	6 (2.2%)	3 (2.8%)	2 (25%)		3 (2.7%)	2 (3.4%)	

**Table 2:** Comparison of sleep quality between participants with Internet addiction.

PSQI categories	No Internet addiction	At risk of Internet addiction	Having Internet addiction	Total	*p* value
	Mean ± SD	Mean ± SD	Mean ± SD	Mean ± SD	
Subjective sleep quality	0.96 ± 0.75	1.2 ± 0.85	1.12 ± 0.83	1.05 ± 0.8	0.04
Sleep latency	1.01 ± 0.88	0.99 ± 0.89	1.62 ± 1.18	1.62 ± 1.18	0.240
Sleep duration	0.83 ± 0.59	0.96 ± 0.54	0.87 ± 0.64	0.87 ± 0.64	0.108
Habitual sleep efficiency	0.19 ± 0.54	0.31 ± 0.67	0.75 ± 1.16	0.75 ± 1.16	0.037
Sleep disturbances	0.78 ± 0.48	1.11 ± 0.72	0.8 ± 0.83	0.8 ± 0.83	0.025
Use of sleeping medication	0.17 ± 0.5	0.44 ± 0.71	0.37 ± 0.74	0.37 ± 0.74	<0.001
Daytime dysfunction	0.99 ± 0.94	1.22 ± 1.03	1.37 ± 1.3	1.37 ± 1.3	0.122
Global PSQI index	4.58 ± 2.7	5.97 ± 3.16	6.6 ± 4.77	6.6 ± 4.77	0.026

**Table 3:** Correlations between dimensions of sleep quality and Internet addiction.

Dimensions	1	2	3	4	5	6	7	8	9
(1) Internet addiction	1								
(2) Subjective sleep quality	0.191^*∗∗*^	1							
(3) Sleep latency	0.129^*∗*^	0.399^*∗∗*^	1						
(4) Sleep duration	0.119^*∗*^	0.218^*∗∗*^	0.105^*∗*^	1					
(5) Habitual sleep efficiency	0.186^*∗∗*^	0.218^*∗∗*^	0.189^*∗∗*^	0.296^*∗∗*^	1				
(6) Sleep disturbances	0.169^*∗*^	0.436^*∗∗*^	0.279^*∗∗*^	0.028	0.163^*∗*^	1			
(7) Use of sleeping medication	0.203^*∗∗*^	0.391^*∗∗*^	0.194^*∗∗*^	0.134^*∗∗*^	0.164^*∗∗*^	0.309^*∗∗*^	1		
(8) Daytime dysfunction	0.188^*∗∗*^	0.532^*∗∗*^	0.252^*∗∗*^	0.076	0.187^*∗∗*^	0.382^*∗∗*^	0.354^*∗∗*^	1	
(9) Global PSQI index	0.291^*∗∗*^	0.771^*∗∗*^	0.628^*∗∗*^	0.351^*∗∗*^	0.429^*∗∗*^	0.550^*∗∗*^	0.481^*∗∗*^	0.622^*∗∗*^	1

^*∗*^Significant at 0.01 (2-tailed). ^*∗∗*^Significant at 0.05 (2-tailed).

## Data Availability

The datasets used and/or analysed during the current study are available from the corresponding author upon reasonable request.
